# Health Tract, No. 155

**Published:** 1863-07

**Authors:** 


### PHYSIOLOGICAL ITEMS.

During the year 1861, fifteen hundred dead bodies were examined at the hospital in Vienna, of which Professor Rokitansky has direction, to ascertain the causes of death. The most prevalent diseases were:

| | | |
|---|---|---|
| 255 Consumption, | 120 Pneumonia, | 101 Cancer, |
| 178 Typhus Fever, | 105 Puerperal, | 67 Peritonitis, |
| 144 Brain, | 109 Heart. | 57 Dysentery. |

Food.—Fish as food, weight for weight, has very nearly as much solid nutriment as butcher's meat, game, or poultry, while containing a substance called iodine, which is not found in land animals, it has a tendency to correct a scrofulous and consumptive habit. Fishermen, who naturally live largely on fish, are especially strong, healthy, and prolific. In no class are there found larger families, handsomer women, and greater exemptions from human maladies. To what extent these results follow a fish diet is as yet a matter of conjecture. But iodine is the universal remedy up to this time for scrofulous diseases.

Meats contain the most nitrogen; the nitrogenous portions of our food make flesh, and go to supply the wear, and tear, and wastes of the body; these are ultimately passed from the system in the urine. If more nitrogenous food is eaten than is needed to supply these wastes, nature converts it more rapidly into living tissues, which are, with corresponding rapidity, broken down and converted into urine. This is when the food is digested; but when so much is eaten that it can not be digested, nature takes alarm as it were and endeavors to remedy the trouble in one of three ways: The stomach rebels and casts it off in disgust by vomiting; it is worked out of the system by an attack of diarrrhea, or the human beast is made so uncomfortable generally that he can't be still; if he goes to bed he tosses and tumbles half the night; if he don't go to bed he is taken with the fidgets and can't be easy in one position for half a minute at a time, so that, in one way or other, he is compelled to an amount of muscular effort necessary to work off the surplus; and as a further punishment, his appetite is more or less destroyed for several meals afterward. Little or no nitrogen is poured off with the perspiration, breathing, or fæces.

Births.—The having a boy or a girl seems to have been a power reserved in the hands of the great and wise Creator of all. The relative proportion, the world over, legitimate and illegitimate, gives about one hundred and six males to one hundred females. In the maufacturing and agricultural districts in England the proportion is identical, seeming to show that the male race is not diminished by crowded houses, unfresh vegetables, bad vapors, poverty, and the like; but it does seem that luxury, inaction, and brain-labor give two per cent less of boys. The greatest thinkers are less apt to have sons; less apt to have vigorous children; less apt to have children at all; and when they do have them they are more likely to die early. The determining power of sex seems thus far to be in the woman, but involuntarily so; she being adapted, an old man is as likely to have a son as a young one, which is contrary to generally received opinions.

